# Metformin Is a Direct SIRT1-Activating Compound: Computational Modeling and Experimental Validation

**DOI:** 10.3389/fendo.2018.00657

**Published:** 2018-11-06

**Authors:** Elisabet Cuyàs, Sara Verdura, Laura Llorach-Parés, Salvador Fernández-Arroyo, Jorge Joven, Begoña Martin-Castillo, Joaquim Bosch-Barrera, Joan Brunet, Alfons Nonell-Canals, Melchor Sanchez-Martinez, Javier A. Menendez

**Affiliations:** ^1^ProCURE (Program Against Cancer Therapeutic Resistance), Metabolism and Cancer Group, Catalan Institute of Oncology, Girona, Spain; ^2^Girona Biomedical Research Institute (IDIBGI), Girona, Spain; ^3^Mind the Byte, Barcelona, Spain; ^4^Unitat de Recerca Biomèdica, Hospital Universitari de Sant Joan, Institut d'Investigació Sanitària Pere Virgili (IISPV), Rovira i Virgili University, Reus, Spain; ^5^Unit of Clinical Research, Catalan Institute of Oncology (ICO) , Girona, Spain; ^6^Department of Medical Sciences, Medical School University of Girona, Girona, Spain; ^7^Medical Oncology, Catalan Institute of Oncology (ICO) Dr. Josep Trueta University Hospital, Girona, Spain; ^8^Hereditary Cancer Programme, Catalan Institute of Oncology (ICO), Bellvitge Institute for Biomedical Research (IDIBELL) L'Hospitalet del Llobregat, Barcelona, Spain; ^9^Hereditary Cancer Programme, Catalan Institute of Oncology (ICO) Girona Biomedical Research Institute (IDIBGI), Girona, Spain

**Keywords:** metformin, SIRT1, aging, NAD^+^, NAD loss

## Abstract

Metformin has been proposed to operate as an agonist of SIRT1, a nicotinamide adenine dinucleotide (NAD^+^)-dependent deacetylase that mimics most of the metabolic responses to calorie restriction. Herein, we present an *in silico* analysis focusing on the molecular docking and dynamic simulation of the putative interactions between metformin and SIRT1. Using eight different crystal structures of human SIRT1 protein, our computational approach was able to delineate the putative binding modes of metformin to several pockets inside and outside the central deacetylase catalytic domain. First, metformin was predicted to interact with the very same allosteric site occupied by resveratrol and other sirtuin-activating compounds (STATCs) at the amino-terminal activation domain of SIRT1. Second, metformin was predicted to interact with the NAD^+^ binding site in a manner slightly different to that of SIRT1 inhibitors containing an indole ring. Third, metformin was predicted to interact with the C-terminal regulatory segment of SIRT1 bound to the NAD^+^ hydrolysis product ADP-ribose, a “C-pocket”-related mechanism that appears to be essential for mechanism-based activation of SIRT1. Enzymatic assays confirmed that the net biochemical effect of metformin and other biguanides such as a phenformin was to improve the catalytic efficiency of SIRT1 operating in conditions of low NAD^+^* in vitro*. Forthcoming studies should confirm the mechanistic relevance of our computational insights into how the putative binding modes of metformin to SIRT1 could explain its ability to operate as a direct SIRT1-activating compound. These findings might have important implications for understanding how metformin might confer health benefits *via* maintenance of SIRT1 activity during the aging process when NAD^+^ levels decline.

## Introduction

A small molecule capable of targeting aging and delaying the onset of aging-related multimorbidity has the potential to radically amend the way we understand (and practice) modern medicine (1). One such molecule is the biguanide metformin, which, 60 years after its introduction in Europe as a first-line therapeutic for type 2 diabetes (2), could have the potential to prevent multiple aging-related disorders (3–5). Against this background, the TAME (Targeting Aging with Metformin) clinical trial has been designed to evaluate the healthspan-promoting effects of metformin by enrolling patients aged 65–79 years diagnosed with one single age-associated condition, and then assessing the global impact of metformin on a composite outcome including cardiovascular events, cancer, dementia, mortality, and other functional and geriatric endpoints (6). Although the current consensus is that metformin has the ability to target multiple pathways of aging, it is still unclear whether such a capacity reflects downstream consequences of a primary action on a single mechanism or whether it involves direct effects on aging regulators (6).

Metformin has been proposed to exert indirect pleiotropy on core metabolic hallmarks of aging such as the insulin/IGF-1 and AMPK/mTOR signaling pathways (4) downstream of its primary inhibitory action on mitochondrial respiratory complex I. Alternatively, but not mutually exclusive, its capacity to operate as a poly-therapeutic anti-aging agent might involve the direct targeting of the biologic machinery of aging *per se*. A systematic chemoinformatics approach established to computationally predict metformin targets recently revealed that the salutary effects of metformin on human cellular aging might involve its direct binding to core chromatin modifiers of the aging epigenome (7, 8), such as the H3K27me3 demethylase KDM6A/UTX (9–11). The ability of metformin to directly interact with TGF-β1, thereby blocking its binding to TβRII and resulting in impaired downstream signaling (12), is another example of how metformin might exert pleiotropic effects on numerous (TGF-β1 hyperfunction-associated) aging diseases such as organ fibrosis and cancer, without necessarily involving changes in cellular bioenergetics.

SIRT1 is a member of the class III (NAD^+^-dependent) histone deacetylases (HDACs) that mimics most of the metabolic responses to calorie restriction and contributes to enhanced healthy aging, including a reduced incidence of cardiovascular and metabolic diseases, cancer, and neurodegeneration (13–17). The regulation of SIRT1 by metformin is an archetypal example of its ability to indirectly and directly impact the aging process. Because of its enzymatic requirement for NAD^+^, SIRT1 is commonly viewed as a unique energy sensor that couples its function to the NAD^+^/NADH ratio of the cell or organism (18–20). Accordingly, metformin-induced metabolic stress has been shown to induce SIRT1 expression and activity as a downstream consequence of AMPK activation-induced augmentation of cellular NAD^+^ levels (21–24). Although the striking similarity between the pleiotropic effects of metformin and the physiological consequences of SIRT1 activation might merely represent the overlapping metabolic effects of SIRT1 and AMPK activators (25, 26), we are beginning to uncover evidence on the occurrence of energy crisis (i.e., AMPK/mTOR)-independent agonist effects of metformin on SIRT1 activity (27–31). Nonetheless, both the putative molecular interactions on the atomic scale between metformin and SIRT1 and the mechanism of action of metformin as a direct modulator of SIRT1 activity remain elusive.

Here, we performed an *in silico* docking and molecular dynamics (MD) simulation study of the SIRT1-metformin complex coupled to laboratory-based experimental validation, aiming to interrogate the ability of metformin to directly enhance NAD^+^-dependent SIRT1 activity. Our findings present a first-in-class structural basis to understand the behavior of metformin as a direct SIRT1-activating compound.

## Materials and methods

### Computational modeling of the human SIRT1 protein

To provide *in silico* insights into the binding pattern of metformin with SIRT1, we employed eight different crystal structures of the human SIRT1 protein, namely 4KXQ, 4IF6, 4ZZJ, 4ZZI, 4ZZH, 4I5I, 5BTR, and 4IG9. 4KXQ, and 4IF6 represent the heterodimeric (chains A and B), closed conformation of SIRT1 bound to adenosine-5-diphosphoribose (APR) (32). 4ZZJ represents the heterodimeric (chains A –SIRT1 and B –p53), open conformation of SIRT1 bound to small molecule sirtuin-activating compounds (STATCs) such as the non-hydrolyzable NAD^+^ analog carbaNAD (carba nicotinamide adenine dinucleotide) or to the carboxamide SIRT1 inhibitor 4TQ (33). 4ZZI represents the monomeric (chain A), open conformation of SIRT1 bound to the carboxamide SIRT1 inhibitors 4TQ and 1NS, whereas 4ZZH represents the monomeric (chain A), open conformation of SIRT1 bound to the carboxamide SIRT1 inhibitor 4TO (33). 4I5I represents the dimeric (chains A and B) conformation of SIRT1 bound to NAD or, alternatively, to the carboxamide SIRT1 inhibitor 4I5 (34). 5BTR represents the heterotrimeric (chains A, B, and C –SIRT1 and D, E, and F –p53), closed conformation of SIRT1 bound to resveratrol (35). Finally, 4IG9 represents a quaternary complex of SIRT1 with no bound ligand (32).

### Docking calculations

All docking calculations were performed using *Itzamna* and *Kin* (www.mindthebyte.com), classical docking and blind-docking software tools. The above mentioned protein structures from RCSB Protein Data Bank (https://www.rcsb.org) were directly employed for docking calculations using the cavities defined by crystallographic ligands where available. Two runs were carried out for each calculation to avoid false positives.

### Molecular dynamics simulations

Docking post-processing allowing conformational selections/induced fit events to optimize the interactions were performed via short (1 ns) MD simulations using NAMD version 2.10 over the best-docked complexes, which were selected based on the interaction energy. The Ambers99SB-ILDN and the GAFF forcefield set of parameters were employed for SIRT1 and metformin, respectively. The GAFF parameters were obtained using Acpype software, whereas the SIRT1 structures were modeled using the leap module of Amber Tools. Simulations were carried out in explicit solvent using the TIP3P water model with the imposition of periodic boundary conditions via a cubic box. Electrostatic interactions were calculated by the particle-mesh Ewald method using constant pressure and temperature conditions. Each complex was solvated with a minimum distance of 10 Å from the surface of the complex to the edge of the simulation box; Na^+^ or Cl^−^ ions were also added to the simulation to neutralize the overall charge of the systems. The temperature was maintained at 300 K using a Langevin thermostat, and the pressure was maintained at 1 atm using a Langevin Piston barostat. The time step employed was 2 fs. Bond lengths to hydrogens were constrained with the SHAKE algorithm. Before production runs, the structure was energy minimized followed by a slow heating-up phase using harmonic position restraints on the heavy atoms of the protein. Subsequently, the system was energy minimized until volume equilibration, followed by the production run without any position restraints.

### Binding free energy analysis

Molecular Mechanics/Generalized Borne Surface Area (MM/GBSA) calculations were performed to calculate the alchemical binding free energy (ΔG_bind_) of metformin against SIRT1. MM/GBSA rescoring was performed using the MMPBSA.py algorithm within AmberTools. The snapshots generated in the 1 ns MD simulation were imputed into the post-simulation MM/GBSA calculations of binding free energy. Graphical representations were prepared using PyMOL program and PLIP version 1.3.0.

### Interaction analysis

The predicted binding site residues of metformin to SIRT1 were defined using evidence-based interaction analyses of known SIRT1 activators/ inhibitors with well-defined binding residues.

### SIRT1 enzymatic assay

The effects of metformin on SIRT1 activity were assessed using the SIRTainty™ Class III HDAC Assay (Cat. #17-10090, Millipore) and the Epigenase™ Universal SIRT1 Activity/Inhibition Assay Kit (Cat. # P-4027, Epigentek), as per the manufacturers' instructions. In the former assay, purified SIRT1 enzyme, β-NAD, acetylated peptide substrate, metformin, and nicotinamidase enzyme were combined and incubated for 30 min. During this time the acetylated peptide substrate is deacetylated by SIRT1 and produces nicotinamide. In a secondary reaction, the nicotinamidase enzyme converts nicotinamide into nicotinic acid and free ammonia (NH${}_{3}^{+}$). To generate a signal for readout, a proprietary developer reagent is added and the signal is read (420_ex_/460_em_ nm) using a fluorescent plate reader. In the latter assay, an acetylated histone SIRT1 substrate is stably coated onto microplate wells; active SIRT1 binds to the substrate and removes acetyl groups from the substrate and the amount of SIRT1-deacetylated products, which is proportional to the enzyme activity, can be measured using a specific antibody. The ratio or amount of deacetylated product, which is proportional to the enzyme activity, is fluorometrically measured by reading the fluorescence at 530_ex_/590_em_ nm. Metformin, phenformin, and buformin (Sigma-Aldrich Ltd.) were added from aqueous stock solutions, and proguanil (Sigma-Aldrich Ltd.) from stock solutions in DMSO.

## Results

### Molecular docking and molecular dynamics simulation analyses of metformin with SIRT1

**Figure 1:**
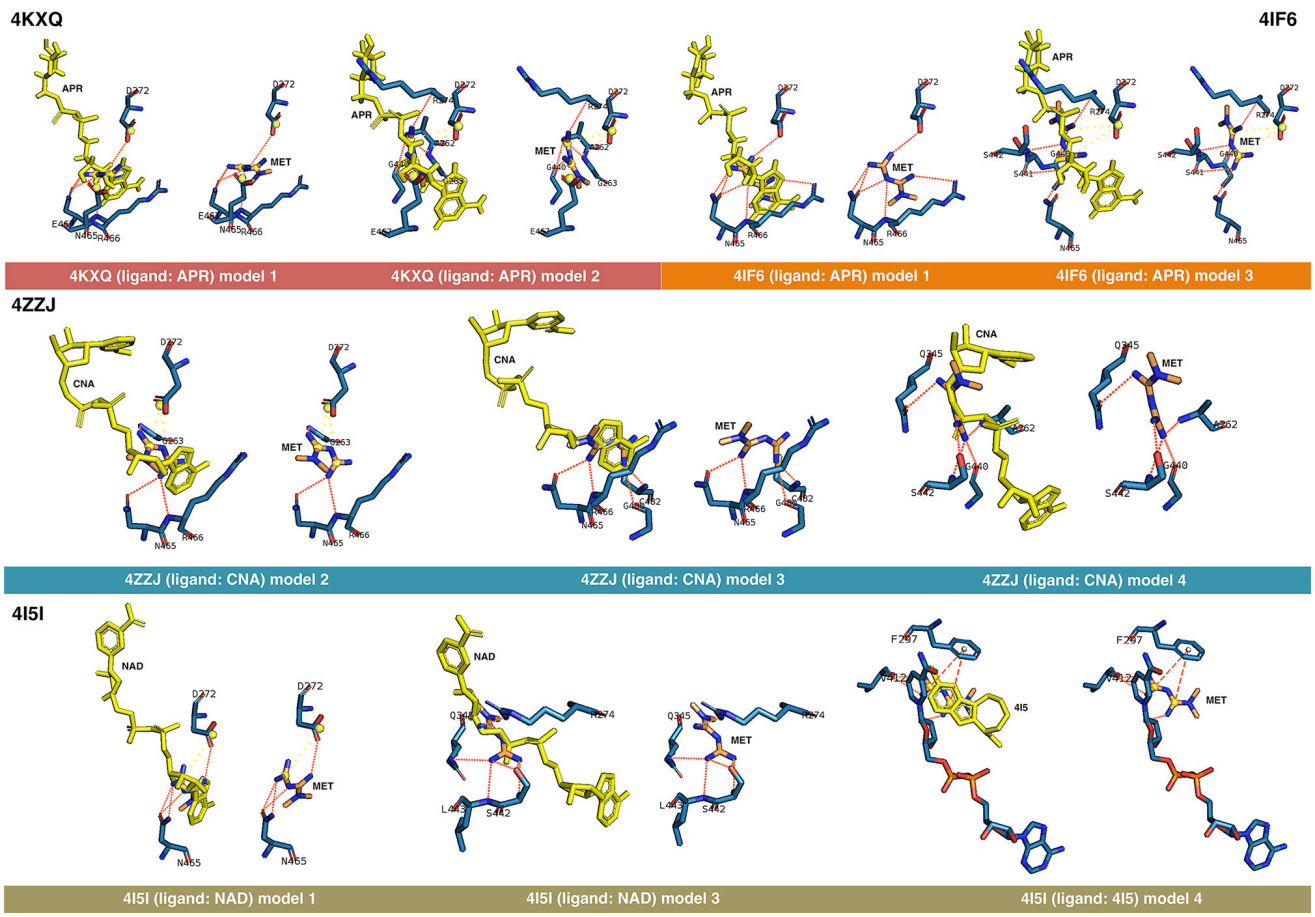
Rigid docking study of the metformin-binding mode to the APR, CNA, NAD^+^, and 4I5 binding pockets of SIRT1. Figure shows in sticks all the pharmacophoric interaction residues involved in the *in silico* binding of metformin to the APR, CNA, NAD^+^, and 4I5 binding pockets of SIRT1, using PLIP. The main residues involved in silibinin interaction with the protein backbone are shown in black; the residue numbers shown correspond to the original PDB file numbering.

**Figure 2:**
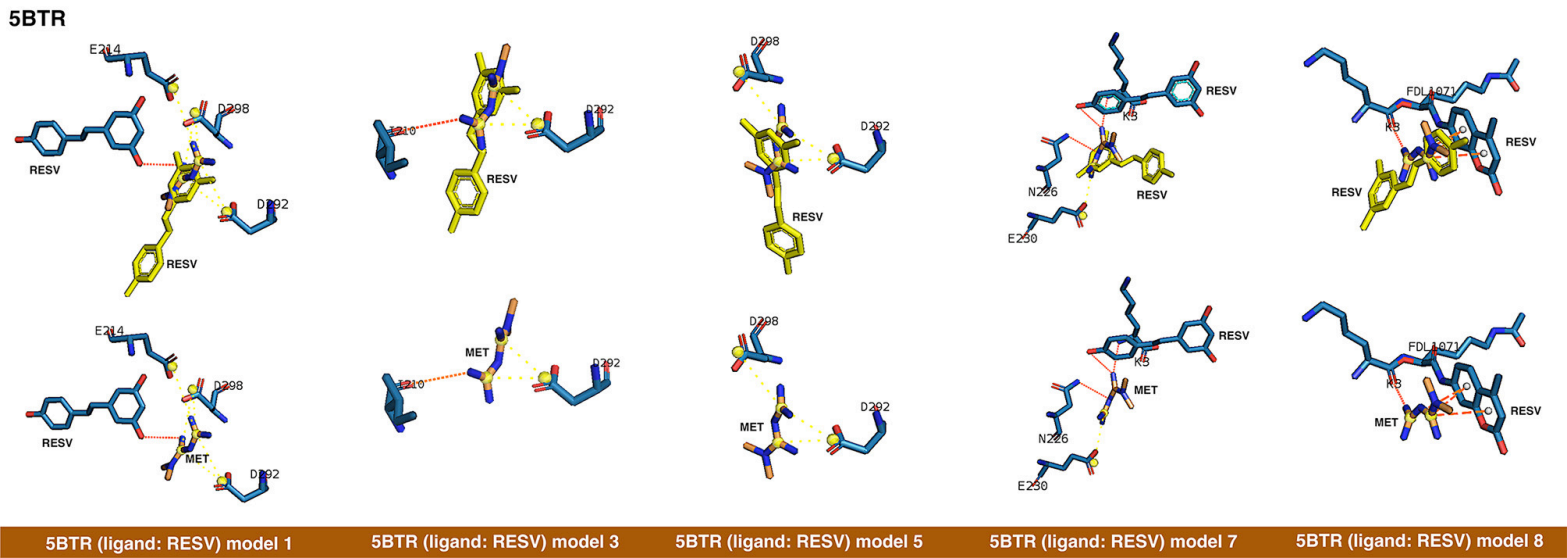
Rigid docking study of the metformin-binding mode to the resveratrol (RESV) binding pocket of SIRT1. Figure shows in sticks all the pharmacophoric interaction residues involved in the *in silico* binding of metformin to the RESV binding pocket of SIRT1, using PLIP. The main residues involved in silibinin interaction with the protein backbone are shown in black; the residue numbers shown correspond to the original PDB file numbering.

First, rigid docking calculations were performed over the cavities defined by the crystallographic ligands in the 4KXQ, 4IF6, 4ZZJ, 4ZZI, 4ZZH, 4I5I, and 5BTR structures (Figures 1, 2). In the case of the ligandless 4IG9 structure, we performed blind docking calculations involving cavity searching and docking calculations over the found cavities. After simulations, we selected more than one model conformation of metformin to cover all the possible binding models within the crystallographic binding poses of the ligands.

**Figure 3:**
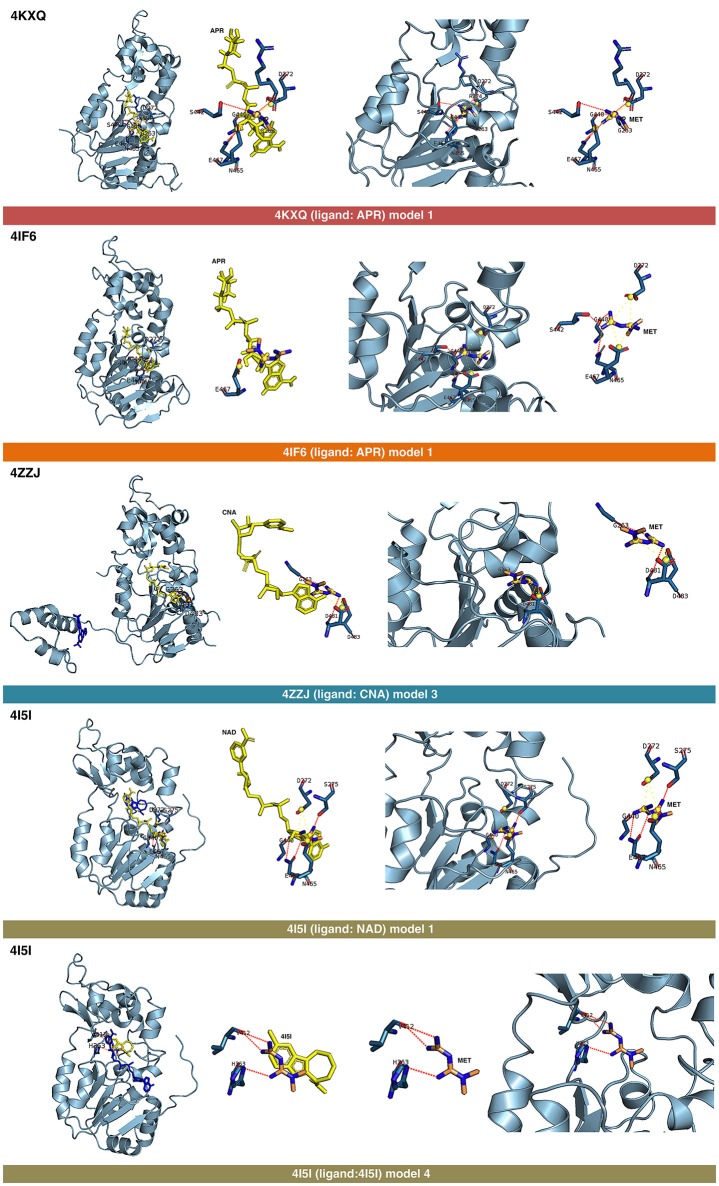
Self-docking poses under molecular dynamics simulations modeling the metformin binding mode to the APR, CNA, NAD^+^, and 4I5 binding pockets of SIRT1. Overall structure and views of the interaction between metformin and the APR, CNA, NAD^+^, and 4I5 binding pockets of SIRT1. The coordinating residues are numbered.

**Figure 4:**
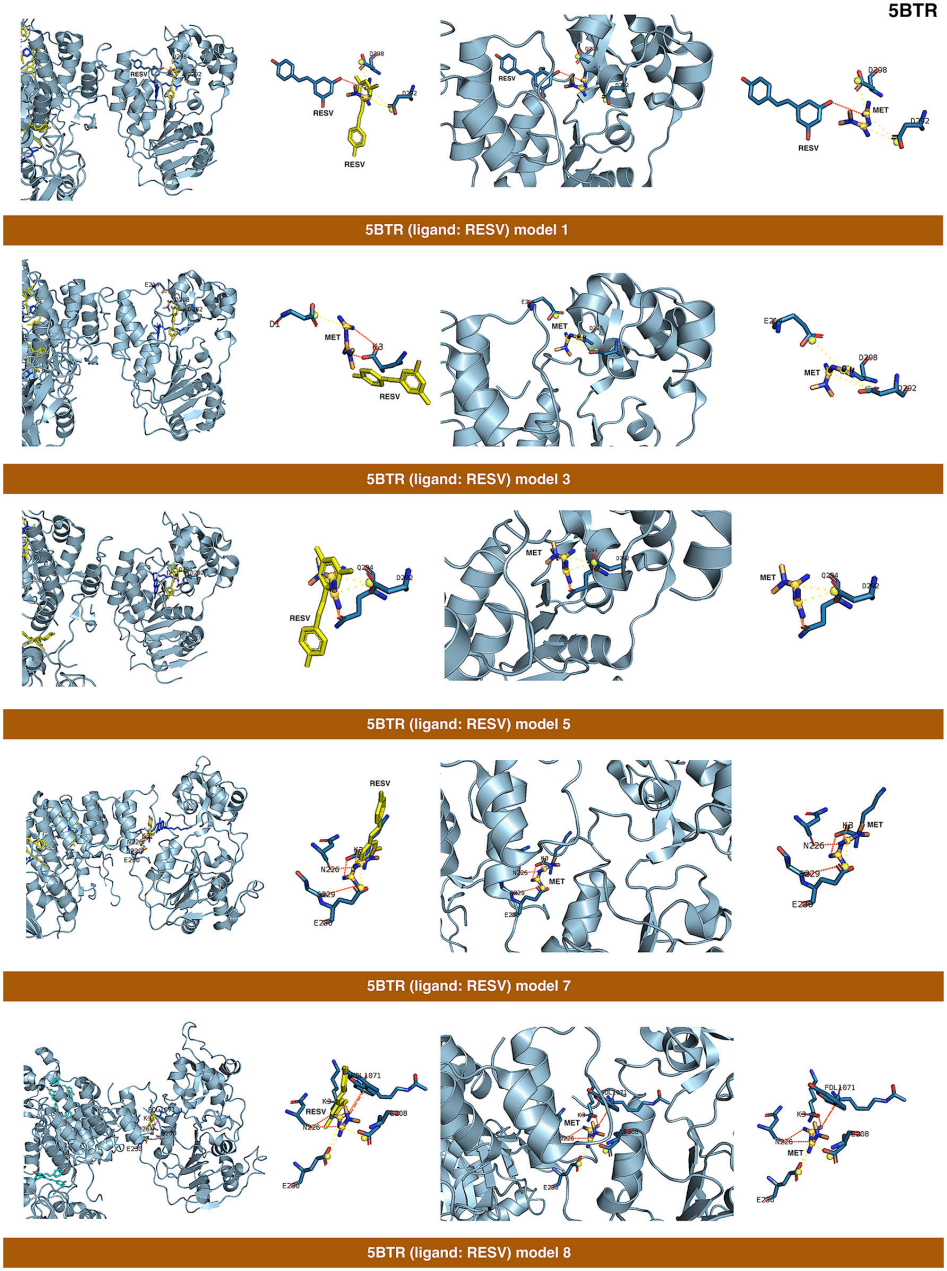
Self-docking poses under molecular dynamics simulations modeling the metformin binding mode to the resveratrol (RESV) binding pocket of SIRT1. Overall structure and views of the interaction between metformin and the RESV binding pockets of SIRT1. The coordinating residues are numbered.

The binding energies obtained from the rigid docking calculations, which were run twice to avoid false positives, are summarized in Table 1. This approach predicted the ability of silibinin to directly bind all the above crystal structures of human SIRT1, with binding energy values up to −5.0 kcal/mol for the crystal structure 4I5I. It should be acknowledged that the predicted *in silico* capacity of metformin to poorly interact with SIRT1, with rather high binding energies, could be explained by the small size of metformin and by docking calculations performed against cavities that, in most cases, are biased toward the ligand to which the target structure is co-crystallized. To add protein flexibility to the analysis and to test the stability of the selected metformin-target complexes, we carried out short MD simulations of 1 ns to filter out poorly interacting poses. We then performed MM/GBSA calculations (36) to estimate the free energy of the binding of metformin to biological macromolecules such as SIRT1. This estimation of ligand-binding affinities takes into consideration the dynamic nature of SIRT1 and it is therefore more reliable to provide a realistic view of metformin binding affinity than rigid docking estimations (Figures 3, 4). The energies obtained following MM/GBSA rescoring calculations over MD simulations are summarized in Table 1, with the best model highlighted in green. From 30 models of metformin-SIRT1 interactions, 11 of them (which are highlighted in green in Table 1) were found to maintain their predicted interacting residues in their corresponding docking poses.

**Table 1:** Docking binding energies and MM/GBSA-based energy rescoring calculations over MD simulations of metformin against SIRT1.

**PDB ID**	**Ligand**	**Model**	**Binding Energy**	**MM/GBSA energy**
			**(kcal/mol)**	**(kcal/mol)^b^**
			**R0/R1^a^**
4KXQ	APR	1	−4.0/−4.6	−18.6175
		2	−3.9/−3.8	−14.6640
		3	−3.3/−2.5	−13.8421
		4	−3.1/−2.0	−4.6926
4IF6	APR	1	−4.2/−4.2	−14.3340
		2	−4.4/−3.8	−12.7689
		3	−3.7/−3.8	−10.4075
		4	−3.1/−2.1	−13.2829
4ZZJ	4TQ	1	−2.3/−2.0	−0.8082
	CNA	2	−3.6/−3.4	−17.8281
		3	−3.5/−4.0	−25.1540
		4	−3.4/−3.4	−21.7529
4ZZI	4TQ	1	−2.1/−2.2	−2.2537
	1NS	2	−4.6/−3.2	−19.0828
4ZZH	4TO	1	−2.1/−1.7	−4.1866
4I5I	NAD	1	−5.0/−5.0	−13.4730
		2	−4.9/−4.9	−5.9833
		3	−4.4/−4.3	−14.8859
	4I5	4	−4.6/−3.7	−16.9806
5BTR	STL-A	1	−3.6/−3.6	−20.8897
	STL-B	2	−3.2/−3.1	−11.2383
		3	−2.9/−2.5	−26.9390
	STL-C	4	−3.2/−3.0	−11.0961
		5	−3.1/−3.2	−16.5834
	STL-D	6	−3.5/−3.7	−10.7575
		7	−3.5/−3.5	−23.6198
	STL-E	8	−3.4/−3.2	−25.0726
	STL-F	9	−3.4/−3.4	−18.9041
4IG9		1	−3.9/−3.9	−2.7150
		2	−4.4/−4.4	−8.3935

*The more negative the binding energy, the more plausible the interaction*.

a *Each docking calculation was performed twice (R0 and R1) to avoid false positives*.

b *Energy obtained after MM/GBSA calculations*.

*Green, best model per target; Yellow, models better maintaining the binding mode in docking and MD studies*.

### Analysis of the binding mode of metformin to SIRT1

The best binding energies of metformin to SIRT1 using rigid docking calculations were predicted to occur when employing the model 1 in the 4KXQ and 4IF6 crystal structures, which highly resemble each other. A detailed analysis of the metformin-binding mode to 4KXQ and 4IF6 predicted the interaction of metformin with the same group of amino acids in both SIRT1 crystal structures, namely D272, G440, S442, N465, and E467.

When evaluating the binding mode of metformin to the open conformation of the heterodimer 4ZZJ, which has two crystallographic ligands (carbaNAD and 4TQ), we observed that metformin was predicted to share one interacting residue (G263) with those predicted in the 4KXQ crystal structure. It is noteworthy that the carbaNAD structure exhibits a reasonable similarity to APR, which is the crystallographic ligand present in 4KXQ and 4IF6. Even though there were no other matching residues, the other predicted interactions suggested a common binding site for 4KXQ and 4IF6, which can be explained in terms of the large size of the cavity in which the interaction could take place, the small size of metformin as a ligand, and the dynamic nature of the protein. When focusing on the crystallographic ligand 4TQ, which is placed at the N-terminal domain (NTD) of 4ZZJ, we predicted a very low interaction energy following MM/GBSA analyses, which can be explained in terms of the exposure of the NTD region and the lack of predicted interacting residues nearby. Therefore, metformin is not predicted to bind the NTD region in the open state of SIRT1.

The monomeric 4ZZI and 4ZZH crystallographic structures contain the ligands 4TQ and 4TO, respectively, at the NTD region of SIRT1. As above predicted for 4ZZJ, we failed to predict any putative interaction of metformin at the NTD region. However, it should be noted that good binding energies were predicted for the crystallographic ligand 1NS, which is placed in a position that is opposed to the cavity occupied by 4TQ and 4TO and, accordingly, we predicted some residues with which metformin could interact with at the 1NS cavity. To better understand this difference, we performed an alignment using 4I5I as a template, finding that 1NS was placed near the terminal benzene ring of the SIRT1 cofactor NAD and the 4I5 cavity. This a region where metformin is predicted to correctly bind according to the results obtained when employing the 4I5I crystallographic structure (see below).

The monomeric conformation of 4I5I contains NAD and 4I5 as crystallographic ligands. When focusing on the NAD binding site, the model 1 predicted a binding mode equivalent to that predicted by the model 1 in 4KXQ and 4IF6, with a good binding energy. Indeed, the predicted interacting residues were shared with those predicted in the model 1 of 4KXQ and 4IF6, namely D272, G440, N465, and E467. When focusing on the 4I5-binding site, it should be noted that the mechanism of action of 4I5 involves a displacement of NAD from its natural site, as it places near the terminal benzene ring of NAD. Interestingly, the predicted interacting residues of metformin were different to those predicted when employing 4KXQ and 4IF6, but similar to those predicted when evaluating metformin binding to the 1NS cavity at 4ZZI. Moreover, the MM/GBSA-based energy binding of metformin at the 4I5 site was reasonably good (−16.9806 kcal/mol), similar to that for 1NS (−19.0828 kcal/mol; Table 1).

The closed conformation of SIRT1 represented by 5BTR with resveratrol as a crystallographic ligand also contains p53 peptides, as in the case of 4ZZJ. Following a detailed analysis of the putative binding modes and predicted residues interactions, we concluded that metformin models 1 for chain A, model 3 for chain B, and model 5 for chain C were placed over the same binding pocket of resveratrol and, importantly, exhibited good binding energies (−20.9987, −26.9390, and −16.5834 kcal/mol, respectively; Table 1). It should be noted that in the case of the model 1 for chain A, an extra resveratrol ligand appears and interacts with metformin, as resveratrol was another residue within the cavity. Good interaction energies were also predicted for chains D (model 7, −23.6198 kcal/mol) and E (model 8, −25.0726 kcal/mol), which represent the same resveratrol ligand. A detailed evaluation of the binding mode of metformin predicted a shared interaction in both models involving N226, E230, and K3 (a residue from p53), thereby suggesting that metformin might bind the closed conformation of SIRT1 at the resveratrol-binding cavity.

The binding mode of metformin to the 4IG9 crystal structure of SIRT1 required a careful and detailed analysis. Following the blind docking calculations, we selected the two models that seemed to better place in the NAD-binding site, which was identified upon structural overlapping. Despite the low interaction energies predicted by MM/GBSA (Table 1), a comprehensive analysis of the interacting residues confirmed the accuracy of the selected cavities and models. Metformin was predicted to move from the docking binding area to a better position near the NAD^+^-binding site. Interestingly, at the end of each MD simulation, metformin was predicted to interact with those residues that seemed to be relevant for defining the binding mode of metformin to SIRT1. The model 1 predicted that the interacting residues after blind docking were R274, F297, and V412. However, following the MD simulation, the residues predicted to be involved in the metformin-binding mode were D292, Q294, and F414. It should be noted that the interacting residues D292 and Q294 were shared also with the binding mode of metformin on the chain C of 5BTR, with D292 emerging as a key residue involved in the metformin-binding mode to the 5BTR crystal. In the model 2 of 4IG9, the sole interacting residue predicted after blind docking was D348. Following MD simulation, however, the residues predicted to be involved in the metformin-binding mode were A262, P271, D272, and F273, with D272 as a key residue involved in the metformin-binding mode to 4KXQ, 4IF6, and 4I5I. Once again, this suggests metformin's capacity to bind not only the inhibitor pocket but also the cofactor cavity of SIRT1.

The displacement of metformin observed when using the ligandless 4IG9 crystal structure of SIRT1 was found to take place also in the model 3 of the 4KXQ crystal, in which the predicted interacting residues in the metformin-binding mode after blind docking were A262, R274, Q345, H363, G440, and S441. By contrast, after MD simulation, the predicted residues were D272, G440, N465, and E467 (i.e., the same group of residues predicted to be involved in the model 1 of 4KXQ crystal). The fact that three of the models that fail to maintain the pose (i.e., model 3 in the 4KXQ crystal, and models 1 and 2 in the 4IG9 crystal) finally move to a better binding site seems to validate the binding modes of metformin observed in other SIRT1 crystal structures.

### Metformin directly enhances SIRT1 enzymatic activity

**Figure 5:**
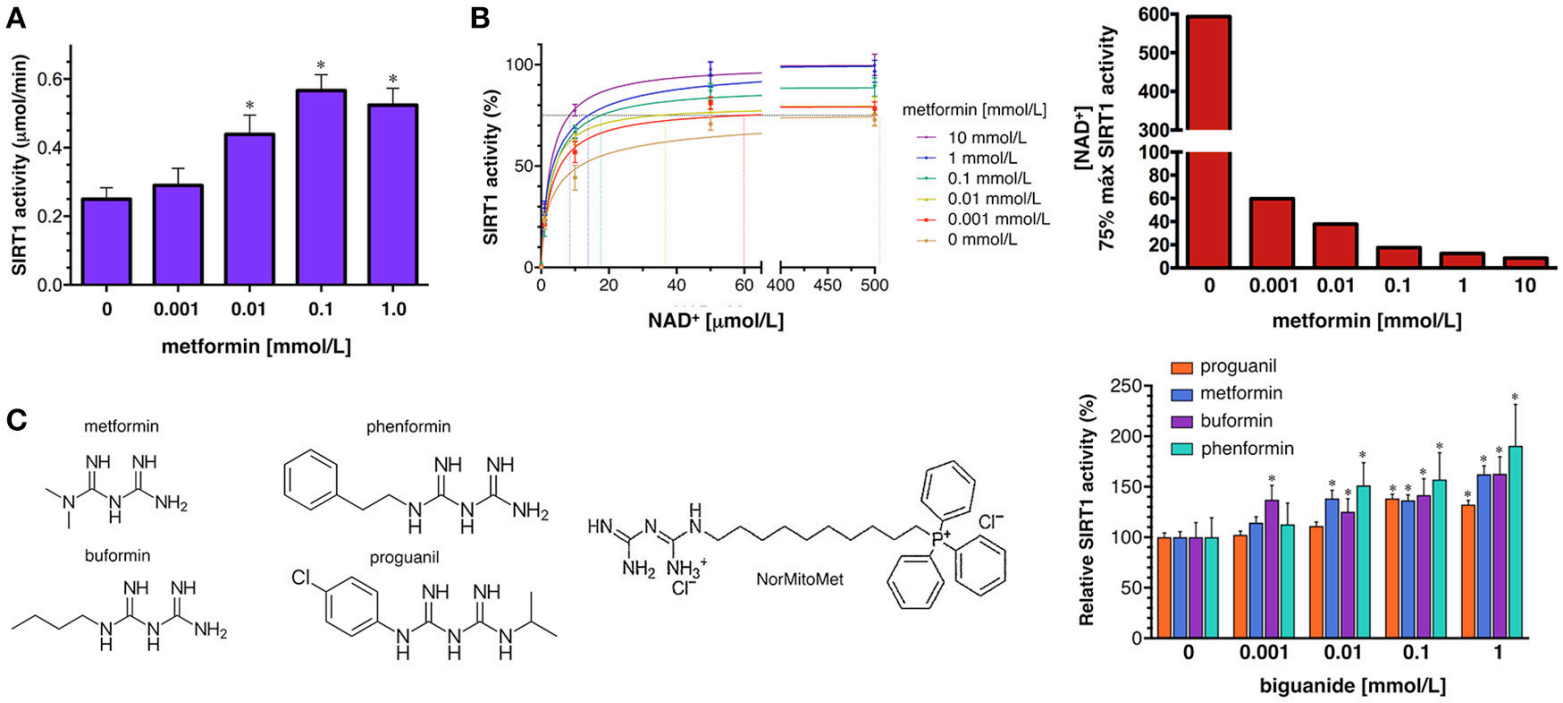
Effects of metformin on the enzymatic activity of SIRT1. **(A)** Dose-response analyses of the effects of graded concentrations of metformin on the activity of SIRT1 using the SIRTainty assay. *Columns* and *error bars* represent mean values and S.D., respectively. Comparisons of means were performed by ANOVA; *P* < 0.01 were considered to be statistically significant (denoted as *). Data points are presented as mean ± SD; three technical replicates per *n*; *n* = 2 biochemical replicates **(B)*** Left*. Human recombinant SIRT1 enzyme was incubated with graded concentrations of NAD^+^ and indicated metformin concentrations in a cell-free system using the Epigenase™ Universal SIRT1 Activity/Inhibition Assay Kit (Fluorometric). Data points are presented as mean ± SD; three technical replicates per *n*; *n* = 2 biochemical replicates. Points are connected by best-fit lines using the Michaelis-Menten model (GrahPad Prism software). *Right*. NAD^+^ concentrations needed to achieve 75% of the maximal SIRT1 activity in the absence or presence of graded concentrations of metformin. **(C) ***Left*. Structural formulas of the compounds with the biguanide moiety highlighted in red. *Right*. Human recombinant SIRT1 enzyme was incubated with 10 μmol/L NAD^+^ in the absence or presence of graded concentrations of biguanides as in **(B)**. Data points are presented as mean ± SD; three technical replicates per *n*; *n* = 2 biochemical replicates.

To confirm the ability of metformin to directly enhance SIRT1 activity, we first used the SIRTainty™ Class III HDAC Assay, which employs nicotinamidase to measure nicotinamide generated upon cleavage of NAD^+^ during SIRT1-mediated substrate deacetylation, and provides a direct assessment of SIRT1 activity. The production of nicotinamide during the 30 min that the acetylated peptide substrate is acted on by SIRT1 was dose-dependently increased by the concomitant presence of graded concentrations of metformin until a saturating plateau level of SIRT1 activity was reached at 1 mmol/L metformin (Figure 5A).

To characterize further how metformin might directly regulate SIRT1 functioning under different NAD^+^ concentrations in a cell-free system, we used the Epigenase™ Universal SIRT Activity/Inhibition Assay Kit. The activation curves of recombinant SIRT1 functioning under different NAD^+^ concentrations in the absence or presence of metformin are shown in Figure 5B. Treatment with graded concentrations of metformin significantly reduced the K_M_ for NAD^+^ while the V_max_ of SIRT1 was slightly increased (up to 30%) in the presence of the highest concentration of metformin tested (10 mmol/L). The metformin-induced leftward-shift of the SIRT1 activation curve, was more evident when evaluating the concentration of NAD^+^ (in terms of relative K_M_) required to achieve of the maximal SIRT1 activity in the presence of metformin, which was increased by 70-fold—from 8.5 μmol/L NAD^+^ in the presence of 10 mmol/L metformin to >500 μmol/L in the absence of metformin (Figure 5B). Perhaps more importantly, the ability of metformin to enhance the capacity of SIRT1 to operate at lower NAD^+^ concentrations similarly occurred at physiological/therapeutic concentrations of metformin; thus, metformin concentrations as low as 1 μmol/L were sufficient to reduce by 7-fold the amount of NAD^+^ required to allow a near-maximal activity of SIRT1.

To evaluate whether pharmacologically relevant biguanides might be viewed as a new family of pharmacologically active SIRT1 activators, we re-evaluated the docking binding energies of several metformin-related biguanides including the anti-malarial biguanides proguanil and cycloguanil, the anti-diabetic biguanides phenformin and buformin, as well as norMitoMet, a novel metformin derivative tagged with the mitochondrial vector triphenylphosphonium (TPP^+^) (37) (Table 2). The open conformations of SIRT1 bound to SIRT1 inhibitors (i.e., 4ZZI-4TQ, 4ZZJ-4TQ, and 4ZZH-4TO) yielded the worst energy binding predictions for all the biguanides. The predicted binding behavior of buformin and proguanil was relatively similar across all the cavities, with the exception of 5BTR (STL-E), which appeared as the preferred one for proguanil. Our molecular docking approach was incapable of predicting the binding energy of cycloguanil to cofactor cavity 4KXQ-APR; very poor energy binding energies were also predicted for norMitoMet and the 4I5I-4I5, 4IG9, 5BTR (STL-E), and 5BTR (STL-F), most likely because of its large size. Phenformin emerged as a good SIRT1-interacting candidate among all the biguanides, exhibiting relatively high binding energies across all the SIRT1 cavities tested, especially against those representing the closed conformation of SIRT1 binding. We then selected proguanil, buformin, and phenformin to experimentally validate the computational predictions. Figure 5C shows that SIRT1 activity was augmented in a dose-dependent manner in the presence of different biguanides, with 1 mmol/L phenformin being capable of enhancing the catalytic activity of SIRT1 by 90% when forced to operate at a NAD^+^ concentration as low as 10 μmol/L.

**Table 2:** Docking binding energies of metformin-related biguanides against SIRT1.

**PDB ID**	**Ligand**	**Biguanide**	**Binding Energy (kcal/mol) R0/R1^a^**
4KXQ	APR	Proguanil	−6.8/−6.7
		Cycloguanil	–
		Buformin	−5.5/−5.6
		Phenformin	−7.2/−7.2
		NorMitoMet	−5.6/−5.0
4IF6	APR	Proguanil	−6.2/−6.2
		Cycloguanil	−5.3/−5.3
		Buformin	−5.7/−5.7
		Phenformin	−7.1/−6.9
		NorMitoMet	−5.6/−4.6
4ZZI	4TQ	Proguanil	−3.7/−3.7
		Cycloguanil	−4.7/−4.7
		Buformin	−2.5/−2.6
		Phenformin	−4.5/−4.4
		NorMitoMet	−5.2/−3.8
4ZZI	1NS	Proguanil	−6.9/−6.9
		Cycloguanil	−7.7/−7.7
		Buformin	−5.7/−5.5
		Phenformin	−7.4/−7.2
		NorMitoMet	−9.0/−8.4
4I5I	4I51	Proguanil	−7.3/−6.5
		Cycloguanil	−7.8/−7.3
		Buformin	−6.2/−6.2
		Phenformin	−6.9/−6.8
		NorMitoMet	1.2/1.1
4I51	NAD	Proguanil	−7.3/−6.2
		Cycloguanil	−7.8/−7.4
		Buformin	−6.2/−5.7
		Phenformin	−6.4/−6.4
		NorMitoMet	−3.8/−3.6
4ZZJ	4TQ	Proguanil	−3.9/−3.1
		Cycloguanil	−4.2/−4.0
		Buformin	−2.8/−2.2
		Phenformin	−2.7/−1.9
		NorMitoMet	−5.1/−4.9
4ZZJ	CNA	Proguanil	−7.4/−7.4
		Cycloguanil	−7.3/−7.3
		Buformin	−5.5/−5.6
		Phenformin	−6.9/−6.0
		NorMitoMet	−8.5/−7.5
4ZZH	4TO	Proguanil	−4.1/−4.0
		Cycloguanil	−4.0/−4.1
		Buformin	−3.4/−3.4
		Phenformin	−3.7/−3.7
		NorMitoMet	−3.7/−3.5
4IG9	–	Proguanil	−5.0/−4.9
		Cycloguanil	−5.7/−5.7
		Buformin	−4.3/−4.3
		Phenformin	−5.5/−5.5
		NorMitoMet	−1.4/−1.2
5BTR	STL-A	Proguanil	−6.9/−6.9
		Cycloguanil	−6.9/−6.9
		Buformin	−5.4/−5.5
		Phenformin	−7.2/−7.1
		NorMitoMet	−7.3/−7.3
5BTR	STL-B	Proguanil	−7.3/−6.8
		Cycloguanil	−7.3/−7.3
		Buformin	−5.3/−5.3
		Phenformin	−7.3/−7.3
		NorMitoMet	−6.8/−6.9
5BTR	STL-C	Proguanil	−6.9/−6.9
		Cycloguanil	−7.6/−7.6
		Buformin	−4.8/−4.8
		Phenformin	−7.0/−6.5
		NorMitoMet	−7.3/−7.6
5BTR	STL-D	Proguanil	−7.5/−7.5
		Cycloguanil	−7.5/−7.5
		Buformin	−5.5/−4.9
		Phenformin	−6.9/−6.4
		NorMitoMet	−7.1/−6.9
5BTR	STL-E	Proguanil	−8.5/−8.6
		Cycloguanil	−6.3/−6.3
		Buformin	−5.6/−5.6
		Phenformin	−8.0/−8.0
		NorMitoMet	6.5/4.2

*The more negative the binding energy, the more plausible the interaction*.

a *Each docking calculation was performed twice (R0 and R1) to avoid false positives*.

## Discussion

**Figure 6:**
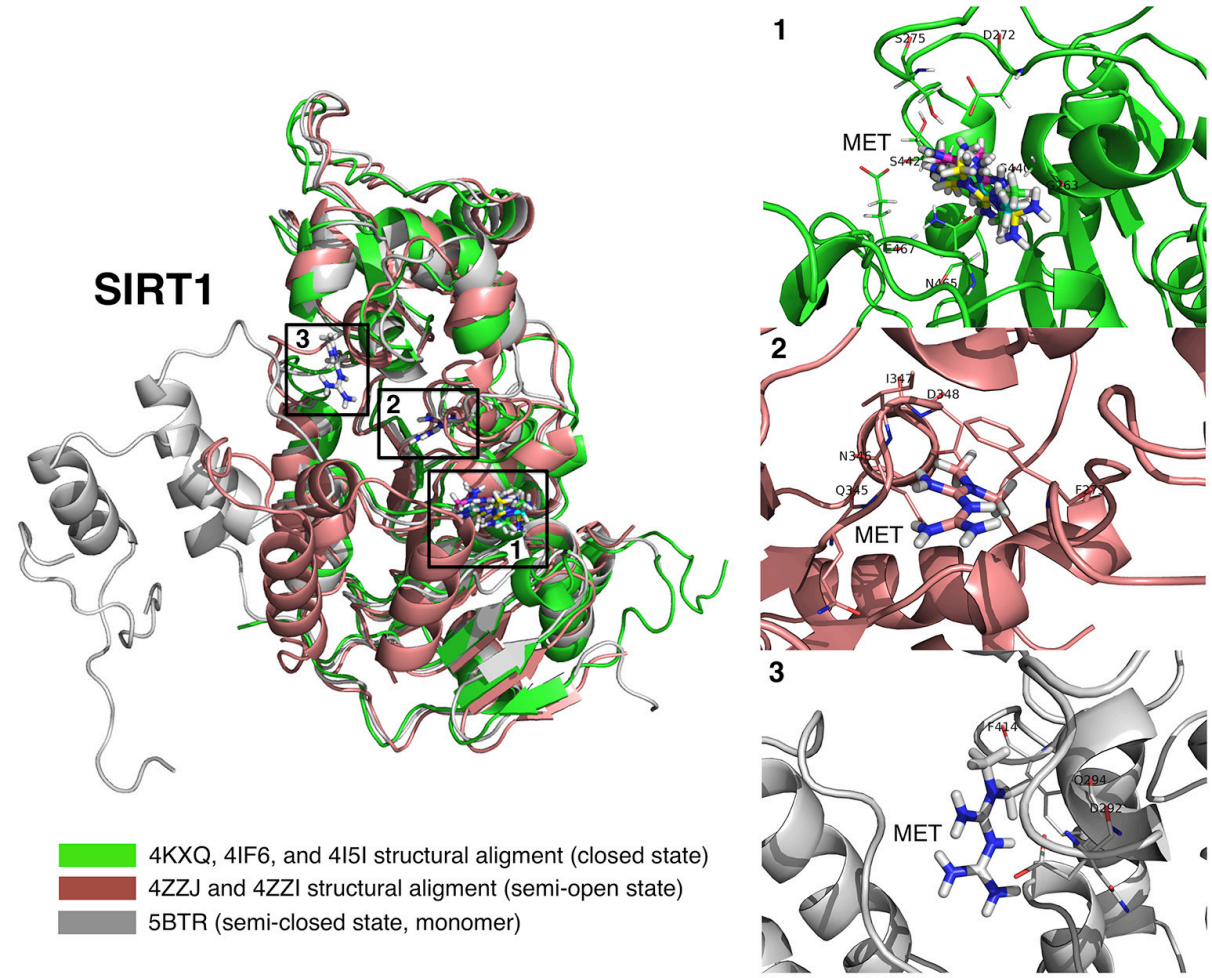
Binding modes of metformin to SIRT1. Global view of the human SIRT1 structure showing the location of the metformin binding sites: (1) metformin poses (4KXQ, 4IF6, 4I5I, and 4ZZJ) at the NAD^+^ binding site, specifically the indole nucleus; (2) metformin poses (4ZZI) at a cavity between the NAD^+^ binding site and the one occupied by the indole derivative (SIRT1 inhibitor) 4I5; and (3) metformin pose at the resveratrol binding pocket at the amino-terminal activation domain of SIRT1.

**Figure 7:**
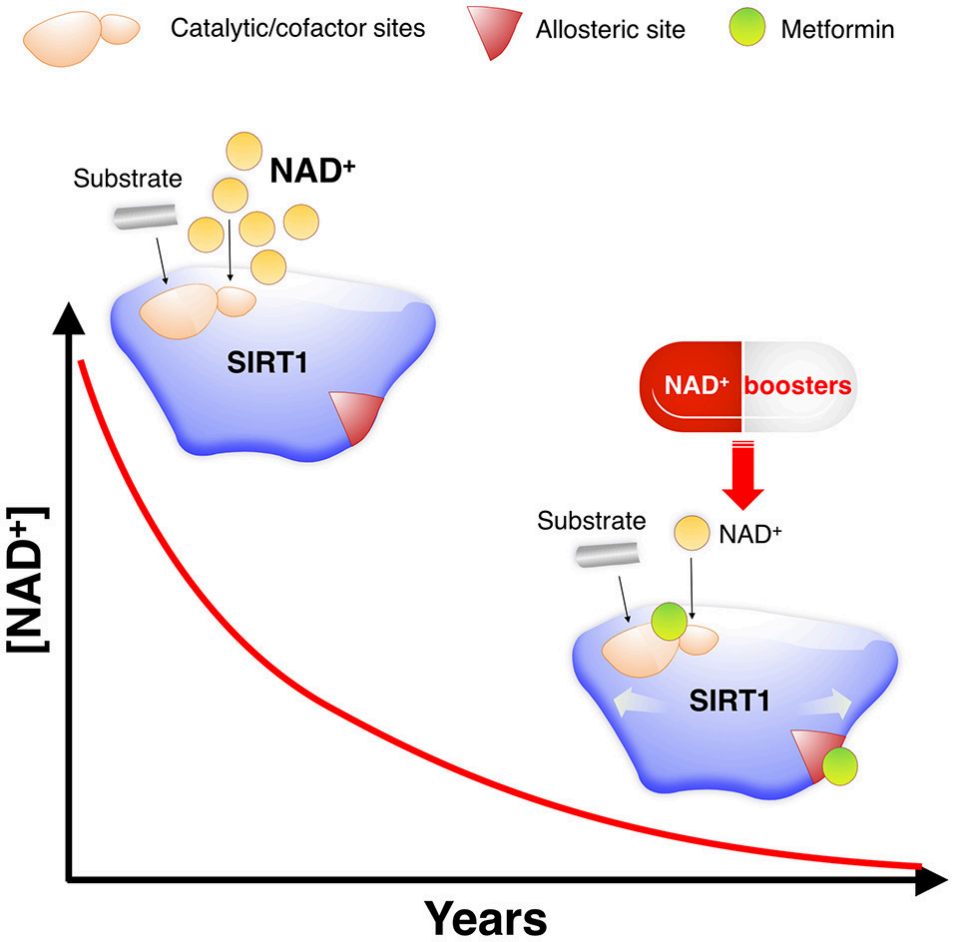
Metformin as a direct SIRT1-activating compound: A new anti-aging role of metformin by modulating NAD^+^-sensing enzymes. SIRT1 activity often declines during aging for reasons other than substrate depletion, namely NAD^+^ decrements. NAD^+^ levels have been described to decrease during aging, mostly due to changes in metabolic pathways leading to NAD^+^ synthesis. Such NAD^+^ deficit is beginning to be viewed as a central mechanism connecting aging and aging-related diseases, including cancer. However, nearly all known STACs target SIRT1 and operate with a limited number of substrates by binding outside of the activate/catalytic site to an allosteric domain of SIRT1 that is not shared with the other sirtuin family members (SIRT2–7) (48). This substrate-dependent, allosteric activation of SIRT1 exerted by the vast majority of STACs cannot compensate for the reduction in NAD^+^ levels. Accordingly, a variety of physiological and pharmacological strategies aimed to boost NAD^+^ levels or inhibit NAD^+^ consumption is being rapidly pursued for nutraceutical and pharmaceutical development to control SIRT1 activity and thereby achieve healthy benefits (44–46, 49). Given the valuable physiological effects of improving the catalytic efficiency of SIRT1 under NAD^+^ depletion in a substrate-independent manner, a preferred general strategy for activation of sirtuins including SIRT1 would be to lower the K_m_ for NAD^+^. K_m_, which would have a similar activating effect to that of NAD^+^ supplementation, could provide specific activation of sirtuin isoforms, and might be achievable without the need to alter the binding affinity of NAD^+^ (50). Our molecular study of the SIRT1-metformin complex coupled to laboratory-based experimental validation strongly suggests that metformin would functionally mimic NAD^+^ boosters by operating as a direct SIRT1-activating compound that ensures health quality during aging via sensitization of SIRT1 to NAD^+^.

We performed a first-in-class computational study aimed to disentangle the putative binding modes of metformin to the SIRT1 enzyme. Our approach reveals that, whereas metformin is predicted to interact with several pockets of SIRT1 inside and outside the central deacetylase catalytic domain (Figure 6), the net biochemical effect is to improve the catalytic efficiency of SIRT1 when it operates at low NAD^+^ conditions *in vitro* (Figure 7). These findings altogether appear to confirm the ability of metformin to operate as a direct SIRT1-activating compound.

When used at low-millimolar concentrations that are incapable of activating the energy-sensing AMPK/mTOR pathway, metformin was previously shown to operate as a *bona fide* SIRT1 agonist to block Th17 cell differentiation, similar to well-known SIRT1 activators such as resveratrol and SRT1720 (27). The capacity of metformin to operate as a direct pharmacological SIRT1 activator, which was defined by the selective targeting of SIRT1 and not the AMPK/mTOR pathway *in vitro* and *in vivo* by therapeutic doses in mice and humans (27, 38), has been further bolstered by the finding that the combination metformin and leucine allows SIRT1 to operate at lower NAD^+^ concentrations in cell-free systems (28–31). Thus, some of the effects of metformin on SIRT1 activation have been proposed to occur *via* its actions as a direct activator of SIRT1, capable of reducing the K_M_ for NAD^+^. We here confirm that physiological/therapeutic concentrations of metformin mimic the effects of calorie restriction by directly promoting an optimal use of NAD^+^ and improving the reaction speed of SIRT1. Importantly, our computational characterization of the putative binding modes of metformin to the regulatory and catalytic pockets of SIRT1 provides new insights into how metformin might directly enhance NAD^+^-dependent SIRT1 deacetylation activity.

Resveratrol and other STACs have been found to facilitate SIRT1 activation by establishing key molecular interactions within a specific STAC-binding allosteric site located at the NTD of SIRT1 (33, 39, 40). Mutagenesis screenings and crystallographic structure studies have provided some information of the interface governing the allosteric binding of STACs. This includes glutamic acid 230 (E230), which appears to be critical for allosteric stimulation of SIRT1 activity by chemically diverse STACs including resveratrol (40) via formation or stabilization of the activated conformation of SIRT1 (33). In addition, asparagine 226 (N226) and aspartate 292 (D292) appear to directly interact with resveratrol and are crucial for the resveratrol-stimulated SIRT1 activity on the substrate (33, 35). It is noteworthy that some of the best SIRT1-metformin complex conformations and SIRT1-metformin binding sites, in terms of binding energies, took place outside of the active site of SIRT1 but involved those residues ostensibly controlling the common mechanism of SIRT1 regulation by allosteric activators, such as E230, N226, and D292. Our biochemical assays showed that metformin sensitizes SIRT1 activity by left-shifting the response of SIRT1 to NAD^+^, which is characteristic of positive allosteric modulators. Besides sensitization, metformin also produces a small but consistent increase in the maximum response of SIRT1 at saturating doses of NAD^+^, which resembles the estimated intracellular content of NAD^+^ in mammals [200–500 μmol/L, (41–44)]. It is therefore tempting to suggest that a concerted allosteric change might occur between the activation domain and the catalytic domain in SIRT1 bound to metformin, thereby allowing SIRT1 to operate at low NAD^+^ concentrations, which mirrors the NAD^+^ deficits occurring during aging [(45–47); Figure 7). The unforeseen capacity of metformin to interact with the STAC-binding allosteric site of SIRT1, which was predicted to solely occur at the substrate-bound closed state, together with the sensitized NAD^+^-SIRT1 activity curve shifting leftwards in the presence of metformin, strongly suggests an allosteric behavior of metformin toward SIRT1. Nevertheless, we acknowledge that our study did not directly evaluate how the binding of metformin to the very same binding pocket of resveratrol at the amino-terminal activation domain might increase NAD^+^-dependent deacetylation of specific substrates. A model of assisted allosteric activation of SIRT1 activation has been proposed, in which STATCs increased the binding affinity for the substrate and *vice versa* (48, 51). Accordingly, it will be interesting to test whether the activation mechanism by metformin is analogous to that of STATCs, lowering the *K*_*m*_ for the substrate and requiring the region around E230. The use of primary cells reconstituted with activation-defective SIRT1 might clarify whether metformin directly activates SIRT1 through an allosteric mechanism capable of decreasing the dissociation constant for specific substrates, which is a common mode of action of other chemically diverse STACs.

Our *in vitro* discovery that metformin allows SIRT1 to operate efficiently at low concentrations of NAD^+^ might alternatively suggest that metformin operates as a mechanism-based enzyme activating compound (MB-STAC) by targeting (and accelerating) the unique NAD^+^-dependent deacetylation turnover mechanism of SIRT1. Although some information is available regarding mechanism-based sirtuin inhibitors (MB-SI) such as Ex-527 and Sir-Real2 (52–54), very little is known about the mechanistic functioning of putative MB-STACs. It has been postulated that a prerequisite for a given modulator to operate as a MB-STAC is the requirement for co-binding with the SIRT1 substrates NAD^+^ and acetylated peptide. Accordingly, crystal structures of SIRT1:MB-SI complexes have shown that MB-SI occupy the nicotinamide site and a neighboring pocket to contact the ribose of NAD^+^ or of the coproduct 2′-O-acetyl-ADP ribose. Interestingly, whereas metformin was predicted to bind the SIRT1 cofactor/inhibitor catalytic regions regardless of the conformational status of SIRT1, it remains to be clarified whether the predicted interacting residues might alter the binding and orientation of the NAD^+^ cofactor, catalytically required to extract a proton from the activated NAD^+^, or are involved in the capture of the released nicotinamide from NAD^+^ (32). Indeed, it should be acknowledged that metformin was predicted to establish interactions with F414, a residue that has been suggested to interact with NAD^+^ (34) and mediate the interaction of the SIRT1 active site with the substrate peptide (32, 33); with N465, a residue that seems to participate in the establishment of an inhibitor-extended conformation of NAD^+^ that sterically prevents productive binding of substrate (34); and also with F273, a key residue involved in the steric blockade of the binding of NAD^+^ in the active conformation of SIRT1 (34). Perhaps more importantly, metformin was predicted to interact with the C-terminal regulatory segment of SIRT1 bound to the NAD^+^ hydrolysis product APR, a “C-pocket”-related mechanism that appears to be essential for MB activation (55, 56). All these elements could be taken to suggest that at low, therapeutic concentrations, metformin might partially mimic the behavior of MB-SI (e.g., by satisfying the requirement of co-binding with substrates) but possessing additional critical attributes necessary to operate as an MB-STAC, including the ability to modulate the local degrees of freedom of the NAD^+^ cofactor and various intermediates and products in the active site. Correspondingly, it could positively alter the balance of productive vs. non-productive SIRT1:NAD^+^ complexes. Conversely, supraphysiological concentrations of metformin might be predicted to force the NAD^+^ cofactor to adopt an inactive binding mode and/or sterically block substrate binding, thereby behaving as a MB-SI. In this vein, metformin concentrations >50 mmol/L were found to significantly reduce SIRT1 enzymatic activity (data not shown). Moreover, our discovery that other metformin-related compounds containing the biguanide functional group (i.e., two guanidiniums joined by common nitrogen) could enhance also SIRT1 activity highlight the importance of considering the biguanides as a new molecular family of weak to moderate direct activators of SIRT1. An enhanced understanding of the molecular pharmacology and mechanisms of biguanide-SIRT1 interactions might enable the design and investigation of novel, more potent metformin-related compounds as direct SIRT1 activators. Nonetheless, our findings provide mechanistic support for recent clinical initiatives conducted to evaluate advantage of the direct activation of SIRT1 by metformin (28–31, 57).

Future studies should confirm the mechanistic relevance of our *in silico* insights into how the putative binding modes of metformin to SIRT1 could explain its ability to operate as a direct SIRT1-activating compound (Figure 7). These findings might have important implications in understanding how metformin could confer health benefits via maintenance of SIRT1 when NAD^+^ levels decline during the aging process.

## Author contributions

EC and SV examined the chemoinformatics data, performed the enzymatic assays, and critically read the manuscript. LL-P, MS-M, and AN-C performed virtual profiling, docking and molecular dynamics calculations, and Molecular Mechanics-Generalized Born/Surface Area scoring. SF-A, JJ, BM-C, JB-B, and JB provided intellectual insight and essential materials necessary for the study. JM conceived the idea, directed the project, and wrote the manuscript.

### Conflict of interest statement

The authors declare that the research was conducted in the absence of any commercial or financial relationships that could be construed as a potential conflict of interest.
